# Regnase-1 in cDC1 controls T cell priming and shapes the dynamics of experimental autoimmune encephalomyelitis

**DOI:** 10.3389/fimmu.2025.1725702

**Published:** 2026-01-05

**Authors:** Xingyu Rong, Hai Wang, Shintaro Muraoka, Takuya Uehata, Masanori Yoshinaga, Tsuneyasu Kaisho, Osamu Takeuchi

**Affiliations:** 1 Department of Medical Chemistry, Graduate School of Medicine, Kyoto University, Kyoto, Japan; 2 Key Laboratory of Breast Cancer in Shanghai, Department of Breast Surgery, Precision Cancer Medicine Center, Fudan University Shanghai Cancer Center, Shanghai, China; 3 Industry-Government-Academia Collaboration Promotion Headquarters, Wakayama Medical University, Wakayama, Japan

**Keywords:** autoimmune disease, conventional type I dendritic cells, inflammation, post-transcriptional regulation, Regnase-1

## Abstract

**Introduction:**

Multiple sclerosis is a chronic inflammatory demyelinating disease of the central nervous system (CNS), in which various immune cells contribute to disease progression, yet the role of dendritic cells (DCs) remains incompletely understood. The RNA-binding protein Regnase-1 plays an important role in regulating immune cell function, but its function in DCs, particularly conventional type 1 DCs (cDC1), has not been defined.

**Methods:**

We investigated the role of Regnase-1 in cDC1 and its impact on experimental autoimmune encephalomyelitis (EAE) on *Regnase-1*^fl/+^ *Xcr1*^-Cre+^ and control mice.

**Results:**

Reduced Regnase-1 expression in cDC1 enhanced pro-inflammatory gene expression and increased their capacity to activate T cells. In the EAE model, *Regnase-1*^fl/+^ *Xcr1*^-Cre+^ mice displayed accelerated inflammatory progression during the acute phase, accompanied by increased infiltration of Th1 cells and activated CD8^+^ T cells in the CNS. Subsequently, *Regnase-1*^fl/+^ *Xcr1*^-Cre+^ mice showed accelerated recovery, together with increased frequencies of central memory CD8^+^ T (Tcm) cells.

**Discussion:**

Our study reveals the complex role for cDC1 with reduced Regnase-1 expression in inflammatory regulation, exacerbating inflammatory responses during the acute phase, while facilitate recovery. This dual role highlights Regnase-1 in cDC1 as a critical regulator that balances inflammation and immune memory.

## 1 Introduction

Multiple sclerosis (MS) is a chronic inflammatory demyelinating disease of the central nervous system (CNS) that causes progressive neurological disability, particularly in young adults (1, 2). Experimental autoimmune encephalomyelitis (EAE) is a classical mouse model that reproduces autoreactive lymphocyte infiltration, demyelination, and axonal injury, and serves as a tool to study MS immuno-pathology (3, 4). CD4^+^ T helper cells, especially Th1 and Th17 subsets, play central pathogenic roles in EAE by producing pro-inflammatory cytokines such as IFN-γ, IL-17A, and GM-CSF (5–8). Meanwhile, CD8^+^ T cells also contribute to the disease progression, by simultaneously producing IFN-γ and TNF-α, exerting cytotoxic effects against oligodendrocytes and neurons (9–12). The activation of both CD4^+^ and CD8^+^ T cells depend on the initial stimulation provided by antigen-presenting cells (APCs) (13–15). Among APCs, dendritic cells (DCs) are the most potent of capturing and presenting myelin antigens, thereby initiating autoreactive T-cell responses (16, 17).

Among DC subsets, conventional type 1 DCs (cDC1) are uniquely specialized for cross-presentation, efficiently activating CD8^+^ T cells and for promoting Th1 polarization through IL-12 production (18, 19). Previous studies have demonstrated that cDC1 play a pivotal role in EAE initiation, as selective depletion of cDC1 using XCR1-targeting CAR-T cells markedly suppresses EAE onset (20). However, the mechanisms by which cDC1 contribute to the progression of EAE remain unclear.

Although the transcriptional regulation in cDC1 has been well characterized, the post-transcriptional regulation of cDC1 in inflammatory conditions is still poorly understood. RNA-binding proteins (RBPs) are key post-transcriptional regulators that control immune cell function and autoimmune responses (21, 22). Regnase-1 (also known as Zc3h12a or MCPIP1) is an RBP that suppresses immune activation by degrading specific target mRNAs (23). Previous studies have mainly focused on the roles of Regnase-1 in macrophages and T cells, demonstrating its essential function in maintaining immune homeostasis and preventing excessive inflammation (24, 25). Notably, we also observed that *Regnase-1^fl/fl^ CD11c-Cre^+^* mice develop a spontaneous autoimmune inflammatory disease, suggesting that Regnase-1is also essential to maintain DC homeostasis (26). However, whether Regnase-1 regulates inflammatory activation and T-cell polarization specifically in antigen-presenting cDC1 remains an important but unexplored question.

Therefore, we hypothesized that Regnase-1 regulates cDC1 inflammatory activation and cytokine-mediated crosstalk with T cells through post-transcriptional control, thereby shaping autoreactive immune responses during CNS autoimmunity. This study aims to define the functional role of Regnase-1 in cDC1 and to elucidate how its loss influences T-cell dynamics in EAE pathogenesis.

## 2 Materials and methods

### 2.1 Mouse studies

*Regnase-1^fl/fl^* mice on a C57BL/6J background have been previously described (25). *Xcr1-Cre^+^*mice on a C57BL/6J background were as described and obtained from RIKEN BioResource Research Center (13). All mice were raised under specific pathogen-free condition and littermate mice were utilized for control. Both male and female mice at 6–10 weeks of age were included in the experiments. All protocols of animal experiments were proved by the Kyoto University Animal Experimentation Committee.

### 2.2 Bone marrow cells isolation and BM-cDC1 differentiation

BM cells were isolated from the femurs and tibias of 6-10-week-old mice. BM cells were obtained by gently flushing and grinding the femurs and tibias in cold PBS to prepare a single-cell suspension, which was subsequently passed through a 40-μm cell strainer to remove debris. Red blood cells were lysed with ACK lysis buffer (ThermoFisher Scientific), and the remaining cells were washed with PBS and resuspended in complete culture medium (RPMI 1640 (Nacalai) supplemented with 10% FBS (ThermoFisher Scientific), 1% penicillin/streptomycin (Nacalai), and 50 μM 2-mercaptoethanol (Nacalai)).

BM-derived cDC1 (BM-cDC1) were generated using a FLT3L-based differentiation protocol (27). Cells were seeded at a density of 5 × 10^6^ cells/mL in 24-well plates and cultured in complete medium containing 100 ng/mL recombinant murine FLT3L (PeproTech) for 9 days. On day 9, non-cDC1 populations were depleted by magnetic bead-based negative selection (anti-PE Microbeads (Miltenyi Biotec)) using antibodies against CD3 (17A2), CD19 (6D5), B220 (RA3-6B2), NK1.1 (PK136), CD64 (X54-5/7.1), F4/80 (BM8), Ly6G (1A8), Ly6C (HK1.4), Ter119 (Ter119), CD317 (927), CD172a (P84). The purity of cDC1 was assessed by flow cytometry using XCR1 (ZET) and CD11c (N418) as surface markers. For LPS stimulation, BM-cDC1 cells were treated with LPS at 100 ng/mL for 1, 2, or 4 hours. For poly(I:C) stimulation, cells were transfected with poly(I:C) at 1 μg/mL using Lipofectamine 2000, and harvested after 2, 4, or 8 hours.

For *in vitro* co-culture experiment, BM-cDC1 were pulsed with SIINFEKL peptide (1 μg/mL; Sigma) for 30 minutes, and subsequently co-cultured with CD8^+^ T cells purified from the spleens of OT-I *Rag2^-/-^* mice supplemented with 5ng/ml recombinant IL-2 (PeproTech). The cDC1 to CD8^+^ T cell ratio was set at 1:5.

### 2.3 RT-qPCR

Total RNA was extracted from cells using TRIzol reagent (Invitrogen) and purified with the RNA Clean & Concentrator kit-5 (Zymo Research) according to the manufacturer’s instructions. Reverse transcription was performed with ReverTra Ace qPCR RT Master Mix with gDNA Remover (Toyobo). The cDNA was amplified using SYBR^®^ Green PCR Master Mix (Applied Biosystems) on a QuantStudio 6Pro or StepOnePlus Real-Time PCR System (Applied Biosystems). Each experiment was performed using at least three independent biological replicates, with each biological sample analyzed in technical triplicate. mRNA expression levels for each sample were normalized to *Actb*. Primer sequences are provided in the **Supplementary Table S1**.

### 2.4 Immunoblot analysis

Cells were collected and lysed in cell lysis buffer (Nacalai) supplemented with Protease Inhibitor Cocktail (Nacalai) to extract total proteins. Equal amounts of protein samples were separated by SDS-PAGE and transferred onto PVDF membranes (Millipore). The membranes were blocked with 5% skim milk for 1 h at room temperature, followed by overnight incubation at 4 °C with the following primary antibodies: anti-Regnase-1 (laboratory-made), and anti-vinculin (Cell Signaling Technology). After washing, membranes were incubated with HRP-conjugated secondary antibodies (Cytiva) for 1 h, and signals were detected using an ECL chemiluminescence system and visualized with an Amersham Imager 680 (Cytiva). For immunoblot at least three biological replicates were analyzed.

### 2.5 RNA-sequencing and bioinformatics analysis

BM-cDC1 were treated with LPS (100 ng/mL) for 4 h, and total RNA was extracted using the NucleoSpin RNA XS kit (Takara Bio) according to the manufacturer’s instructions. Subsequent sample processing and sequencing followed previously described methods (28). RNA integrity was assessed using a Bioanalyzer (Agilent) with the RNA 6000 Pico Kit (Agilent), and only samples with an RNA integrity number (RIN) greater than 7 were used. RNA-seq libraries were prepared with the NEBNext Ultra II Directional RNA Library Prep Kit for Illumina (New England BioLabs) following the manufacturer’s protocol. Sequencing was performed on an Illumina NextSeq 500 using the NextSeq 500/550 High-Output v2 Kit (Illumina). RNA-seq data were quality-controlled and quantified by quasi-mapping to the reference transcriptome using Salmon, followed by aggregation to the gene level based on the gene annotation file. Normalization and differential expression analysis were performed with DESeq2. Significantly differentially expressed genes (DEGs) were defined as those with a log_2_flod change > 0.3 and adjusted p value < 0.05. Identified DEGs were subjected to GO and pathway enrichment analyses.

### 2.6 EAE mouse model

Eight- to ten-week-old wild type (control) and *Reg1^fl/+^Xcr1Cre^+^* mice were immunized with MOG 35–55 peptide (Anaspec) to induce EAE. MOG 35–55 was dissolved in PBS and emulsified in complete Freund’s adjuvant (CFA) containing 10 mg/mL heat-killed *Mycobacterium tuberculosis* H37Ra (Chondrex). A total of 200 μg of MOG 35–55 emulsion was administered subcutaneously at the base of the tail and back. Pertussis toxin (200 ng; List Biological Laboratories, Inc.) was injected intraperitoneally on the day of immunization and again on day 2. From day 7 post-immunization, mice were monitored daily for clinical scores until the experimental endpoint. EAE clinical scores were assessed as follows: 0 = normal; 1 = limp tail; 2 = hind limb weakness; 3 = partial hind limb paralysis; 4 = complete hind limb paralysis; 5 = moribund or death. Both male and female mice are used, and data from both sexes were pooled. Immunization and score evaluation order is randomized. Scoring was performed according to standardized criteria, and genotype information was not explicitly referenced during evaluation to reduce potential bias.

### 2.7 Biological sample collection and preparation of single-cell suspensions

Spinal cord: Spinal cords were digested in HBSS containing 20 U/mL papain (Sigma), 100 μg/mL DNase I (Sigma), and 15 mM HEPES (Nacalai) at 37 °C for 30 min. The digested tissue was filtered through a 70-μm nylon mesh strainer, washed, and immune cells were enriched by density gradient centrifugation using a 30%/70% Percoll gradient.

Spleen and lymph nodes: Single-cell suspensions were prepared by mechanical dissociation and passage through a 70-μm cell strainer. Splenocytes were subjected to red blood cell lysis before use.

Intracellular cytokine staining was performed after stimulating cells with cell stimulation cocktail and protein transport inhibitor cocktail (Thermo Fisher Scientific) in accordance with the manufacturer’s instructions.

### 2.8 Flow cytometry

Single-cell suspensions were washed with MACS buffer (PBS supplemented with 2 mM EDTA and 0.5% BSA) and incubated with anti-mouse purified CD16/CD32 (BD Biosciences) for 10 min on ice to block Fc receptors. Cell viability was assessed using Fixable Viability Dye (FVD, Thermo Fisher Scientific). Cells were then stained with the following antibodies:

Surface markers: CD45.2 (104), CD3 (17A2), CD4 (GK1.5), CD8 (53-6.7), CD44 (IM7), CD62L (MEL114), CD11c (N418), XCR1 (ZET), H-2K^b^ (28-8-6), CD86 (GL-1).

Intracellular cytokines/effector molecules: IFN-γ (XMG1.2), IL-17A (TC11-18H10), TNF-α (MP6-XT22), granzyme B (GB11).

Gating strategy for cDC1 (CD45^+^CD11c^+^Xcr1^+^), CD3^+^ T cells (CD45^+^CD3^+^), CD4^+^ T cells (CD45^+^CD3^+^CD4^+^) and CD8^+^ T cells (CD45^+^CD3^+^CD8^+^) are shown in **Supplementary Figure S1A**.

### 2.9 Statistical analysis

All data were analyzed using GraphPad Prism 10. The specific statistical methods for each group of data are described in detail in the figure legends.

## 3 Results

### 3.1 Regnase-1 reduction in cDC1 enhances proinflammatory gene expression without affecting type I IFN responses

**Figure 1:**
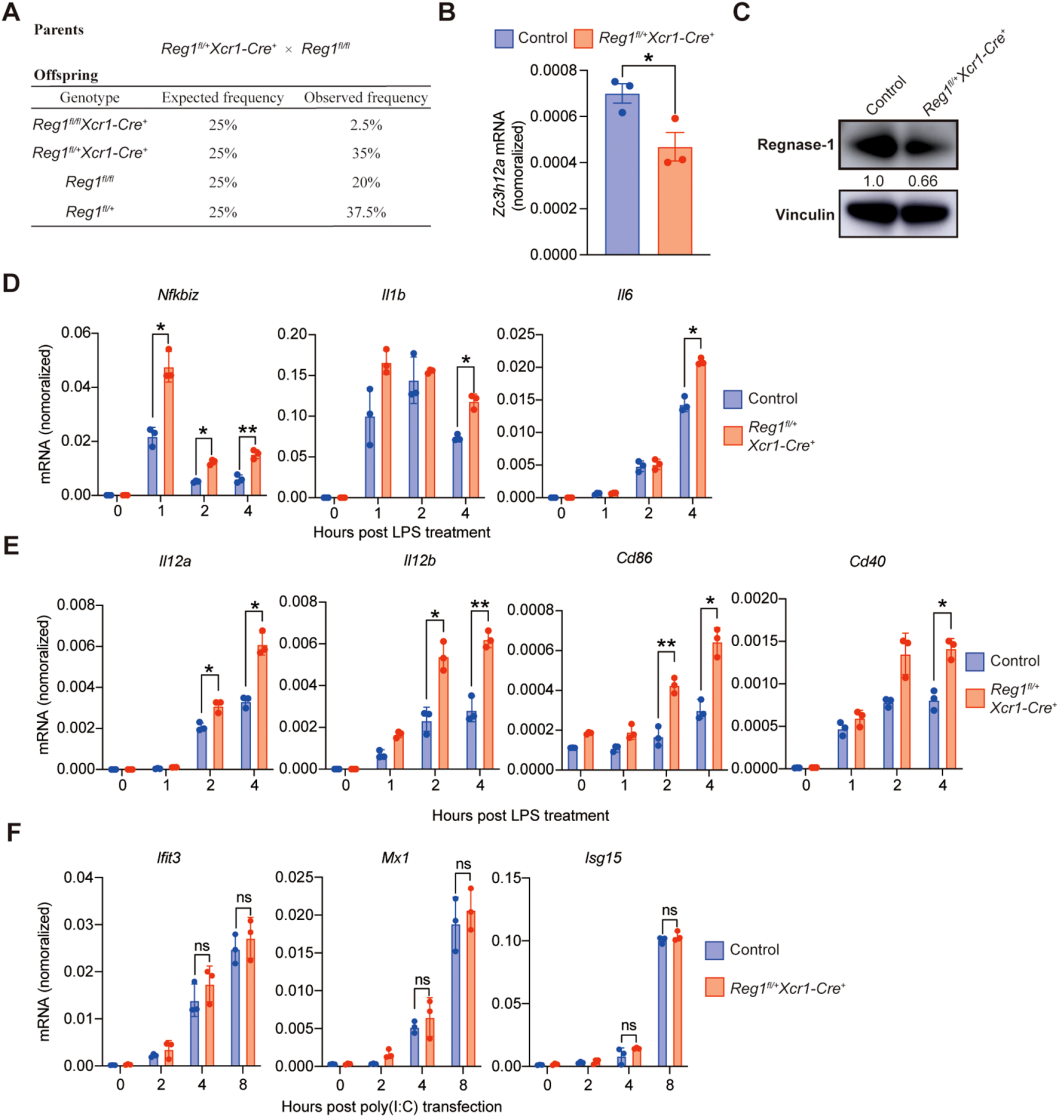
Regnase-1 reduction in cDC1 enhances proinflammatory gene expression without affecting type I IFN responses. **(A)** Breeding strategy and expected versus observed genotype frequencies of offspring derived from *Regnase-1^fl/+^ Xcr1-Cre^+^* × *Regnase-1^fl/fl^* crosses. Genotyping of offspring was performed at 4 weeks of age by tail cutting. **(B)** qPCR analysis of *Zc3h12a* mRNA (encoding Regnase-1) in BM-cDC1 from control (n=3) and *Regnase-1^fl/+^ Xcr1-Cre^+^* mice (n = 3). **(C)** Immunoblot analysis of Regnase-1 protein expression in BM-cDC1 from control and *Regnase-1^fl/+^ Xcr1-Cre^+^* mice. **(D)** qPCR analysis of inflammatory genes in control and *Regnase-1^fl/+^ Xcr1-Cre^+^* cDC1 after LPS stimulation (100 ng/mL) (n = 3). **(E)** qPCR analysis of T cell activation-related genes in control and *Regnase-1^fl/+^ Xcr1-Cre^+^* cDC1 after LPS stimulation (100 ng/mL) (n = 3). **(F)** qPCR analysis of interferon-stimulated genes (ISGs) expression in control and *Regnase-1^fl/+^ Xcr1-Cre^+^* cDC1 following poly(I:C) transfection (1 μg/mL) (n = 3). Data are shown as mean ± s.e.m. Data present biological replicates. Statistical analysis: **(A)** unpaired t test; **(C, E, F)** one-way ANOVA with Tukey-test. Reg1, Regnase-1. *P < 0.05; **P < 0.01; ns, not significant.

To examine the role of Regnase-1 in cDC1, we attempted to generate *Regnase-1^fl/fl^ Xcr1-Cre^+^* mice by crossing *Regnase-1^fl/+^ Xcr1-Cre^+^* mice with *Regnase-1^fl/fl^* mice. Although the offspring of Regnase*-1^fl/fl^ Xcr1-Cre^+^* mice were expected at a Mendelian frequency of 1/4, the actual frequency observed was 1/40 (**Figure 1A**). Considering *Regnase-1^fl/fl^ Xcr1-Cre^+^* mice exhibited poor viability, we used heterozygous *Regnase-1^fl/+^ Xcr1-Cre^+^* mice to investigate the role of Regnase-1 in cDC1. qPCR and immunoblotting analyses confirmed that both Regnase-1 mRNA and protein levels were reduced in BM-cDC1 from *Regnase-1^fl/+^ Xcr1-Cre^+^*mice (**Figures 1B, C**). Upon LPS stimulation, cDC1 from *Regnase-1^fl/+^ Xcr1-Cre^+^* mice exhibited higher expression of *Nfkbiz*, *Il6* and *Il1b* compared with control (**Figure 1D**), indicating enhanced induction of proinflammatory genes in Regnase-1-reduced cDC1. In addition, Regnase-1-reduced cDC1 also showed higher expression of T cell activation-associated genes, including *Cd86*, *Cd40*, *Il12b* and *Il12a* (**Figure 1E**), suggesting augmented antigen-presenting and co-stimulatory capacity. Since Regnase-1 may also effect the regulation of type I IFN responses, we next examined the induction of interferon-stimulated genes (ISGs) after poly(I:C) stimulation. The expression of interferon-stimulated genes (ISGs) such as *Mx1* and *Ifit3* remained comparable between the two groups after poly(I:C) stimulation (**Figure 1F**). Thus, reduction of Regnase-1 in cDC1 selectively enhances proinflammatory gene and T cell stimulation, without affecting ISG induction.

### 3.2 Regnase-1 reduction in cDC1 enhances proinflammatory gene expression and Th1/CD8^+^ T cell priming

**Figure 2:**
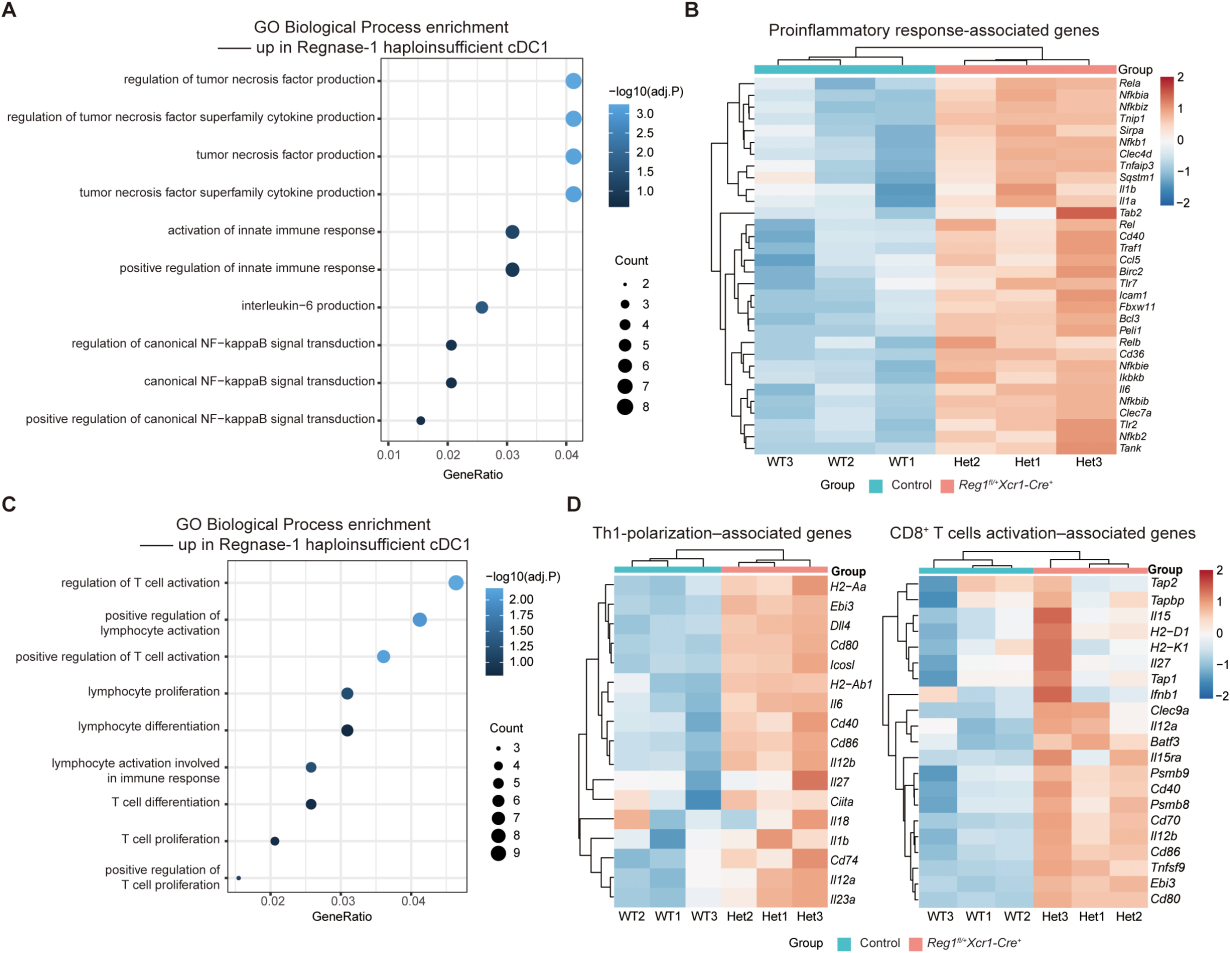
Regnase-1 reduction in cDC1 enhances proinflammatory signaling and Th1/CD8^+^ T cell priming. **(A)** GO enrichment analysis of innate immune response process among differentially expressed genes. Dot size indicates the proportion of genes, and color reflects significance. **(B)** Heatmap of proinflammatory response-associated genes. Blue indicates low expression, and red indicates high expression. **(C)** GO enrichment analysis of T cell activation-related processes among differentially expressed genes. Dot size indicates the proportion of genes, and color reflects significance. **(D)** Heatmaps of Th1 polarization-related genes (left) and CD8^+^ T cell activation-related genes (right). Blue indicates low expression, and red indicates high expression. Data are shown as normalized expression (z-scores). Statistical analysis: DESeq2 with Benjamini-Hochberg FDR control. Reg1, Regnase-1.

To comprehensively define the role of Regnase-1 in cDC1, we compared the transcriptomes of control and *Regnase-1^fl/+^ Xcr1-Cre^+^* cDC1 following LPS stimulation. In cDC1 with reduced Regnase-1 expression, pathways related to inflammatory cytokine production, innate immune response and NF-κB signal transduction were enriched (**Figure 2A**). Consistently, the expression of inflammatory cytokines (*Il1b, Il6, Ccl5, etc.*) and NF-κB-related genes (*Nfkbia*, *Tnfaip3*, *Relb, etc.*) was increased (**Figure 2B**), suggesting an enhancement of proinflammatory responses.

In parallel, pathways related to T cell activation were enriched in *Regnase-1^fl/+^ Xcr1-Cre^+^* cDC1, suggesting enhanced immune-stimulatory ability (**Figure 2C**). Heatmap analysis further revealed elevated expression of genes associated with Th1 polarization (e.g., *Il12b*, *Il12a*, *Cd40*, *Cd86*) and CD8^+^ T cell priming (e.g., *Tap1*, *Tapbp*, *H2-K1*, *Tnfsf9*) (**Figure 2D**).

Together, these findings demonstrate that Regnase-1 reduction in cDC1 promotes proinflammatory gene expression and enhances their capacity to support Th1 and CD8^+^ T cell response.

### 3.3 Reduced expression of Regnase-1 cDC1 exacerbates EAE onset but accelerates recovery

**Figure 3:**
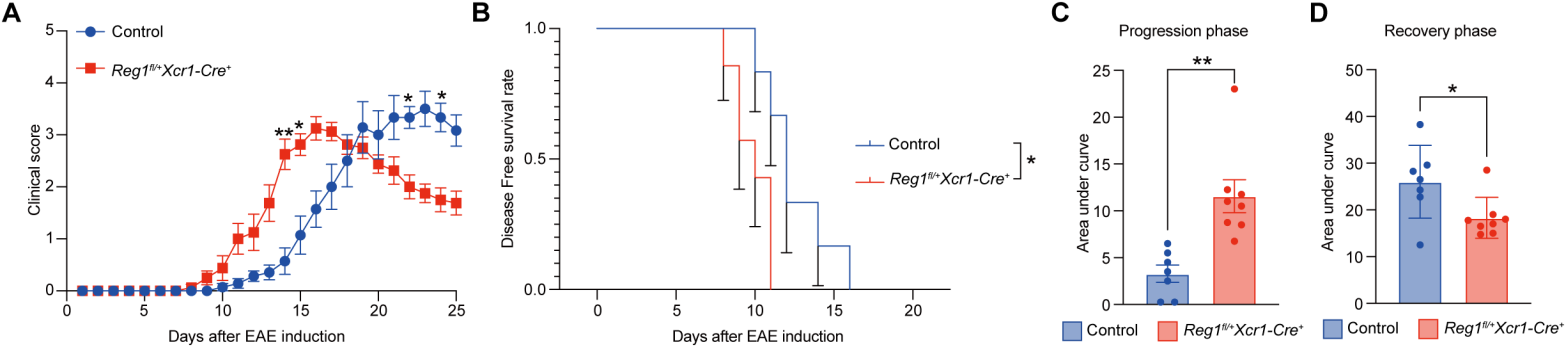
Reduced expression of cDC1 exacerbates EAE onset but accelerates recovery. **(A)** Clinical scores of EAE in control (n=7) and *Regnase-1^fl/+^ Xcr1-Cre^+^* (n=8) mice were monitored daily after EAE induction. **(B)** Kaplan-Meier curves for disease-free survival after EAE induction. Error bars represent sem. **(C)** Quantification of disease severity during the progression phase (day 0-16), calculated by the area under the curve (AUC) of clinical scores. **(D)** Quantification of disease severity during the recovery phase (day 17-25), calculated by the AUC of clinical scores. Data are shown as mean ± s.e.m. Data present biological replicates. Statistical analysis: **(A)** two-way ANOVA (mixed-effects model) with Geisser-Greenhouse correction for repeated measures. Bonferroni’s multiple-comparison test was applied for *post hoc* analysis. **(B)** Mantel-Cox test, **(C, D)** unpaired t test. Reg1, Regnase-1. EAE, Experimental autoimmune encephalomyelitis. *P < 0.05; **P < 0.01.

To determine the effect of Regnase-1 in cDC1 on inflammatory responses *in vivo*, we employed the EAE model. Control and *Regnase-1^fl/+^ Xcr1-Cre^+^* mice were immunized with MOG 35–55 to induce disease. Clinical score curves revealed that *Regnase-1^fl/+^ Xcr1-Cre^+^* mice developed symptoms earlier, with a more rapid rise in clinical scores and an earlier peak compared with controls (**Figure 3A**). Kaplan-Meier analysis further confirmed that these mice exhibited an earlier onset of EAE, with a reduced disease-free survival rate (**Figure 3B**). To quantitatively assess disease dynamics, we calculated the area under the curve (AUC) separately for the progression phase (day 0-16) and the recovery phase (day 17-25). *Regnase-1^fl/+^ Xcr1-Cre^+^* mice showed higher AUC values during the progression phase, reflecting more severe inflammation at early stages (**Figure 3C**).

In contrast, their AUC values were lower during the recovery phase, suggesting a faster resolution from symptoms (**Figure 3D**). Collectively, these results indicate that reduction of Regnase-1 in cDC1 exerts dual effects on the EAE disease course, promoting earlier onset in the initial phase while facilitating accelerated recovery during the later phase.

### 3.4 Reduced expression of Regnase-1 in cDC1 drives peripheral T cell expansion during EAE progression phase

Given that reduced Regnase-1 expression in cDC1 affects both the progression and recovery phases of EAE, we first evaluated the immune status of mice at day 16 (progression phase). Considering that cDC1 primarily activate T cells in the draining lymph nodes (DLNs), we examined the lymph nodes status (**Supplementary Figure S1A**). Under unimmunized conditions, *Regnase-1^fl/+^ Xcr1-Cre*^+^ mice showed no apparent lymph node enlargement compared with controls (**Supplementary Figures S1B, C**), and both the frequency and the cross-presentation capacity of cDC1 were comparable between the two groups in the steady state (**Supplementary Figures S1D, E**). Consistently, T cell numbers in the lymph nodes showed no difference under unchallenged conditions (**Supplementary Figure S1F**). These results indicate that Regnase-1 haploinsufficiency in cDC1 does not induce systemic inflammation.

**Figure 4:**
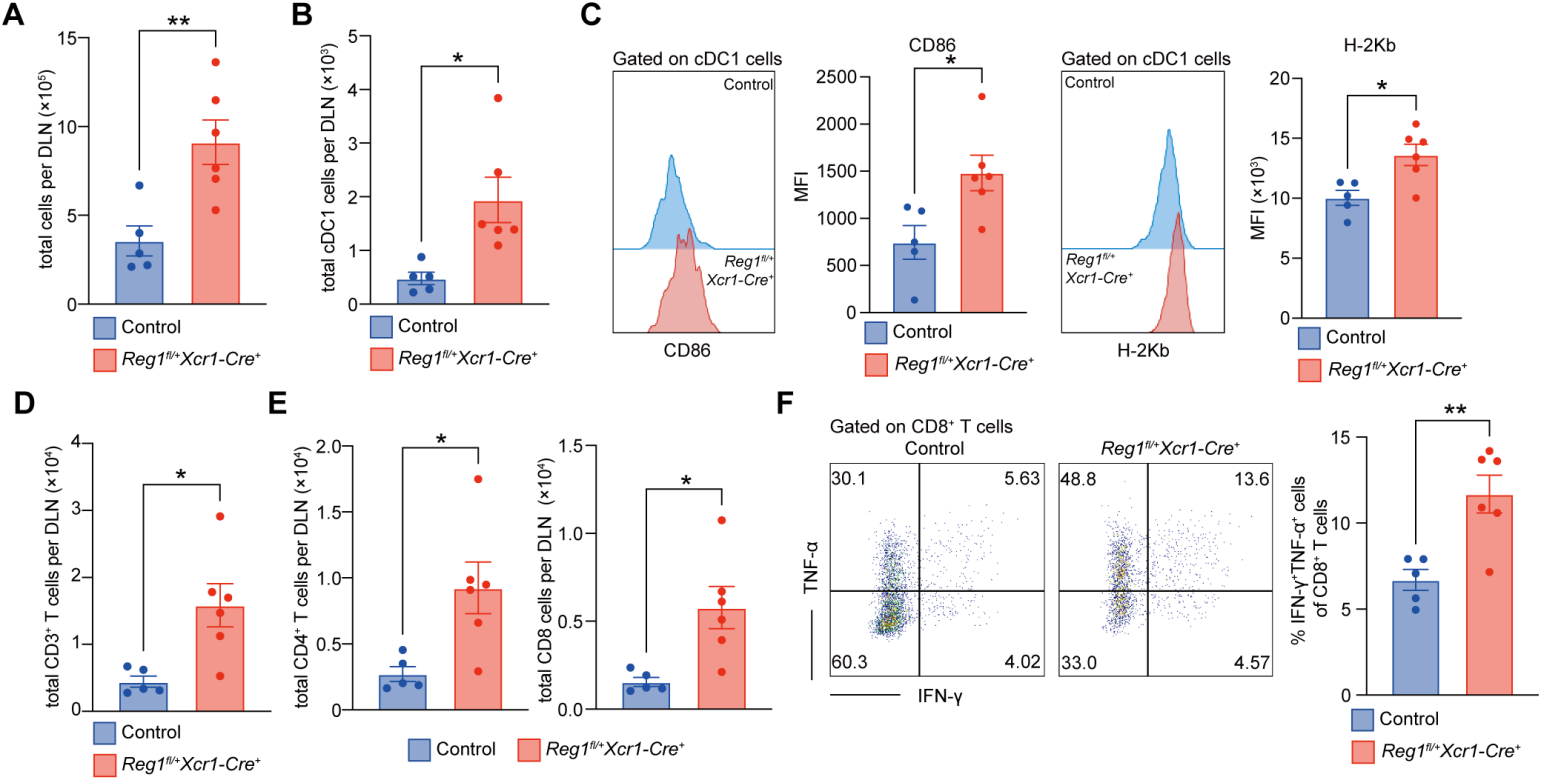
Reduced expression of cDC1 drive peripheral T cell expansion during EAE progression phase. Flow cytometric analysis of DLNs collected at the peak of progression phase of EAE (Day 16) from control (n= 5) and *Regnase-1^fl/+^Xcr1-Cre^+^* (n=6) mice. Gating strategy is shown in **Supplementary Figure S1**. **(A)** Total cell numbers per DLN. **(B)** Numbers of cDC1 cells (CD45^+^CD11c^+^Xcr1^+^) per DLN. **(C)** Expression of CD86 and H-2K^b^ on cDC1. Representative histograms (left) and quantification (right). **(D)** Total numbers of CD3^+^ (CD45^+^CD3^+^) T cells per DLN. **(E)** Total numbers of CD4^+^ (CD45^+^CD3^+^CD4^+^) and CD8^+^ (CD45^+^CD3^+^CD8^+^) T cells per DLN. **(F)** Frequency of IFN-γ^+^ TNF-α^+^ double-positive CD8^+^ T cells among total CD8^+^ T cells. Representative plots (left) and quantification (right). Data are presented as mean ± s.e.m. Data present biological replicates. Statistical analyses were performed using unpaired t test. DLN, draining lymph node; Reg1, Regnase-1. *P < 0.05; **P < 0.01.

We next analyzed DLNs at Day 16 after EAE induction. Both groups exhibited lymph node enlargement, but DLNs of *Regnase-1^fl/+^ Xcr1-Cre^+^* mice were significantly larger than those of controls (**Supplementary Figure S1G**, **Figure 4A**), suggesting stronger immune activation in heterozygous mice. Flow cytometric analysis showed that while the proportion of cDC1 among total immune cells remained similar, their absolute numbers increased (**Figures 4B**, **Supplementary Figure S1H**). Moreover, cDC1 from *Regnase-1^fl/+^ Xcr1-Cre^+^* mice expressed higher levels of CD86 and H-2K^b^ (**Figure 4C**), consistent with enhanced T cell-stimulatory capacity. In parallel, the total numbers of CD3^+^, CD4^+^, and CD8^+^ T cells were elevated in the DLNs of *Regnase-1^fl/+^ Xcr1-Cre^+^* mice (**Figures 4D, E**; **Supplementary Figures S1I, J**). Furthermore, the frequency of IFN-γ^+^ TNF-α^+^ double-positive CD8^+^ T cells was also increased (**Figure 4F**), implying a more activated effector state. In summary, these findings demonstrate that reduced Regnase-1 expression in cDC1 drives T cell expansion and activation in the DLNs during the progression phase of EAE, providing a peripheral foundation for subsequent CNS immune infiltration.

### 3.5 Reduced Regnase-1 expression in cDC1 promotes CD8^+^ T cell infiltration and effector function in the CNS

**Figure 5:**
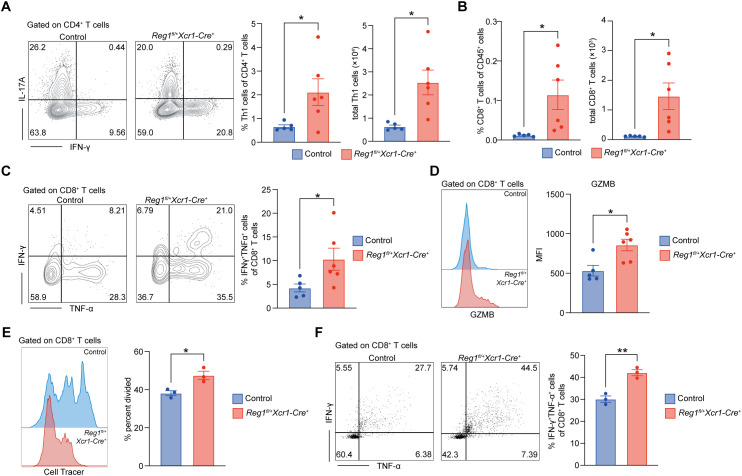
Reduced Regnase-1 expression in cDC1 promotes CD8^+^ T cell infiltration and effector function in the CNS. **(A–D)** Flow cytometric analysis of immune cells infiltrating the spinal cord on Day 16 after EAE induction in control (n=5) and *Regnase-1^fl/+^ Xcr1-Cre^+^* (n=6) mice. **(A)** Th1 cells (CD45^+^ CD3^+^ CD4^+^IFN-γ^+^IL17A^-^) among CD4^+^ T cells, representative plots and quantification of frequency and total number. **(B)** CD8^+^ T cells (CD45^+^CD3^+^CD8^+^) among CD45^+^ cells, percentage and absolute numbers. **(C)** IFN-γ^+^ TNF-α^+^ double-positive CD8^+^ T cells among total CD8^+^ T cells, representative plots and quantification. **(D)** Granzyme B expression on spinal cord-infiltrating CD8^+^ T cells, representative histogram and quantification. **(E, F)***In vitro* co-culture of BM-cDC1 from control (n=3) or *Regnase-1^fl/+^ Xcr1-Cre^+^* (n=3) mice with splenic CD8^+^ T cells isolated from OT-I *Rag2^-/-^* mouse for 48 hours (for CellTracer proliferation test) and 24 hours (for cytokine detection). **(E)** CD8^+^ T cell proliferation assessed by CFSE dilution, representative figure and quantification. **(F)** IFN-γ and TNF-α expression in CD8^+^ T cells, representative dot plots and quantification. Data are presented as mean ± s.e.m. Data present biological replicates. Statistical analyses were performed using unpaired t test. DLN, draining lymph node; Reg1, Regnase-1; BM-cDC1, bone marrow-derived conventional DC1; GZMB, Granzyme B. *P < 0.05; **P < 0.01.

To further explore whether Regnase-1 reduction in cDC1 affects CNS inflammation, we analyzed immune cell infiltration in the spinal cord at the progression phase of EAE (Day 16) (**Supplementary Figure S2A**). Although Th17 cells infiltration did not differ between the two groups (**Supplementary Figure S2B**), *Regnase-1^fl/+^ Xcr1-Cre^+^*mice exhibited a marked increase in both the frequency and total number of Th1 cells in the spinal cord (**Figure 5A**). In addition, the proportion and total number of infiltrating CD8^+^ T cells were significantly higher in *Regnase-1^fl/+^ Xcr1-Cre^+^* mice (**Figure 5B**; **Supplementary Figure S2C**), accompanied by an increased percentage of IFN-γ^+^ TNF-α^+^ double-positive CD8^+^ T cells (**Figure 5C**), indicating enhanced effector function. Furthermore, spinal cord-infiltrating CD8^+^ T cells from *Regnase-1^fl/+^ Xcr1-Cre^+^* mice expressed higher levels of Granzyme B, suggesting elevated cytotoxic activity (**Figure 5D**).

To test whether the enhanced CD8^+^ T cell expansion and response was driven by the heightened activation status of Regnase-1 depleted cDC1, we established an *in vitro* co-culture system (**Supplementary Figure S2D**). BM-cDC1s from *Regnase-1^fl/+^ Xcr1-Cre^+^* or control mice were loaded with SIINFEKL peptide, and then co-cultured with splenic CD8^+^ T cells isolated from OT-I *Rag2^-/-^*mice. Compared with control cDC1s, Regnase-1-reduced cDC1s significantly promoted CD8^+^ T cell proliferation (**Figure 5E**) and increased the frequency of IFN-γ^+^ TNF-α^+^ double-positive cells (**Figure 5F**).

Overall, these findings demonstrate that reduced expression of Regnase-1 in cDC1 enhances their ability to activate CD8^+^ T cells. Regnase-1-reduced cDC1 promotes Th1 and CD8^+^ T cell infiltration and effector activation in the CNS, ultimately driving the rapid progression of EAE.

### 3.6 Reducing Regnase-1 in cDC1 promotes CD8^+^ central memory T cell differentiation during EAE recovery phase

**Figure 6:**
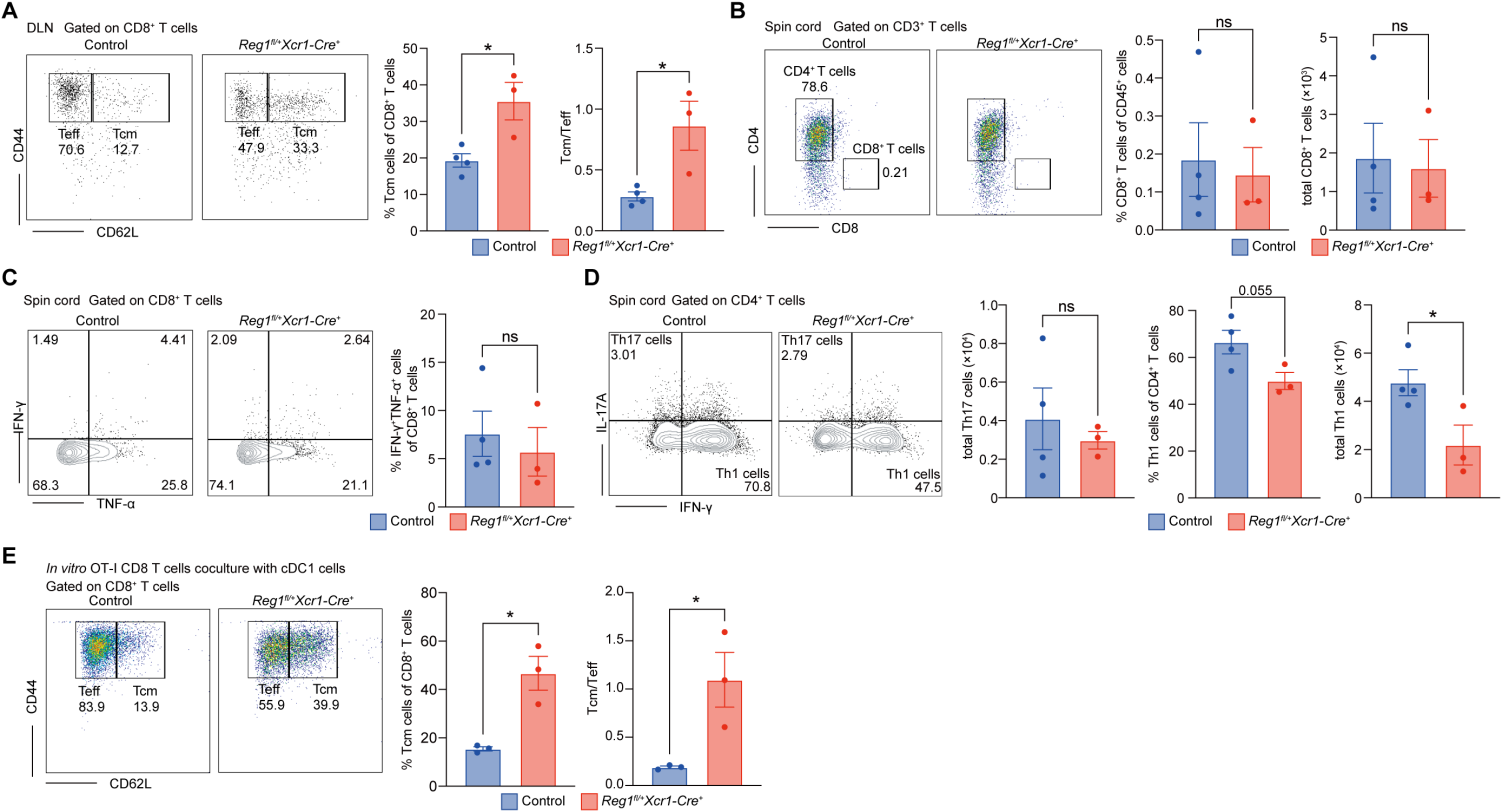
Reducing Regnase-1 in cDC1 promotes CD8^+^ central memory T cell differentiation during EAE recovery phase. **(A)** Flow cytometric analysis of CD8^+^ T cells from DLNs in control (n=4) and *Regnase-1^fl/+^ Xcr1-Cre^+^* mice (n=3) on Day 25 after EAE induction. The proportion of central memory T cells (Tcm; CD44^+^CD62L^+^) and the ratio of Tcm to effector T cells (Teff; CD44^+^CD62L^−^) were quantified. Representative plots and quantification are shown. **(B–D)** Flow cytometric analysis of immune cells infiltrating the spinal cord on day 25 after EAE induction in control (n=4) and *Regnase-1^fl/+^ Xcr1-Cre^+^* mice (n=3). **(B)** Frequency and absolute number of CD8^+^ T cells. Representative plots and quantification are shown. **(C)** Proportion of IFN-γ^+^ TNF-α^+^ double-positive CD8^+^ T cells. Representative plots and quantification are shown. **(D)** Frequency and absolute number of Th1 and Th17 CD4^+^ T cells. Representative plots and quantification are shown. **(E)***In vitro* co-culture assay: BM-cDC1 cells from control (n=3) and *Regnase-1^fl/+^ Xcr1-Cre^+^* (n=3) mice were loaded with SIINFEKL and co-cultured with splenic CD8^+^ T cells isolated from OT-I *Rag2*^−^/^−^ mice for 72 hours. The proportion of Tcm and the Tcm/Teff ratio in CD8^+^ T cells were analyzed. Representative plots and quantification are shown. Data are presented as mean ± s.e.m. Data present biological replicates. Statistical analyses were performed using unpaired t test. LN, draining lymph node; Reg1, Regnase-1; BM-cDC1, bone marrow-derived conventional DC1. *P < 0.05; ns, not significant.

To assess whether Regnase-1 reduction in cDC1 affects the immune response during the recovery phase of EAE, we analyzed T cell profiles in both peripheral lymph nodes and the CNS on day 25 after EAE induction. In the DLNs, *Regnase-1^fl/+^ Xcr1-Cre^+^* mice exhibited a higher proportion of CD8^+^ central memory T cells (Tcm; CD44^+^CD62L^+^) and an increased Tcm/Teffector cells (Teff; CD44^+^CD62L^−^) ratio compared to controls (**Figure 6A**), suggesting that Regnase-1 reduction in cDC1 facilitates CD8^+^ T cells central memory differentiation in the recovery phase.

Meanwhile, the infiltration of CD8^+^ T cells in the spinal cord of *Regnase-1^fl/+^ Xcr1-Cre^+^* mice was no longer elevated (**Figure 6B**), and the frequency of IFN-γ^+^ TNF-α^+^ double-positive CD8^+^ T cells was comparable to the controls (**Figure 6C**), indicating that the previously activated CD8^+^ T cell response had resolved. Analysis of inflammatory CD4^+^ T cells in the CNS showed that Th17 cell numbers in the spinal cord were markedly reduced in both groups compared to the progression phase, with no significant difference between two groups. However, Th1 cell numbers were lower in *Regnase-1^fl/+^ Xcr1-Cre^+^* mice (**Figure 6D**), consistent with a more rapid resolution of central inflammation in the *Regnase-1^fl/+^ Xcr1-Cre^+^* group.

To further investigate whether Regnase-1-reduced cDC1 harbors an enhanced ability to promote CD8^+^ Tcm differentiation, we performed an *in vitro* co-culture assay. BM-cDC1 from control or *Regnase-1^fl/+^ Xcr1-Cre^+^* mice were loaded with SIINFEKL, and co-cultured with splenic CD8^+^ T cells isolated from OT-I *Rag2*^−^/^−^ mice for 72 hours. CD8^+^ T cells stimulated by Regnase-1-reduced cDC1 exhibited a significantly increased proportion of Tcm and a higher Tcm/Teff ratio (**Figure 6E**).

Collectively, these results suggest that Regnase-1 deficiency in cDC1 not only accelerates early CD8^+^ T cell activation and CNS infiltration during the acute phase but also facilitate the remodeling of CD8^+^ T cells toward a central memory phenotype in the recovery phase.

## 4 Discussion

This study reveals that Regnase-1 is a critical regulator of cDC1 in inflammation and autoimmunity. We show that Regnase-1-reduced cDC1 enhances proinflammatory gene expression, which in turn promotes CD8^+^ T cell and Th1 cell responses during the early phase of EAE. Conversely, reducing Regnase-1 in cDC1 facilitates the generation of CD8^+^ Tcm cells during the recovery phase, which may contribute to the disease recovery. These findings delineate a dual role of Regnase-1 in shaping the immune balance in EAE.

Transcriptomic analysis showed that reduced Regnase-1 expression in cDC1 upregulated multiple proinflammatory genes, many of which overlapped with previous identified Regnase-1 targets from our RIP-seq analysis, including *Il6*, *Il1b*, *Nfkbiz* and *Rassf3* (23). These findings suggest that Regnase-1 restrains cDC1 activation by degrading cytokine mRNA and limiting *Nfkbiz* expression, thereby preventing NF-κB-driven *Il12b* transcription. Consequently, Regnase-1 reduction enhances the NF-κB-NFKBIZ-IL-12 signaling axis in cDC1, which may lead to enhanced Th1 and CD8^+^ T-cell priming in EAE.

Garg et al. reported that knockdown of Regnase-1 enhances IL-17–mediated signaling and exacerbates EAE by promoting Th17 differentiation (29). In contrast, *Regnase-1^fl/+^ Xcr1-Cre^+^* mice did not affect Th17 responses, likely reflecting the intrinsic IL-12-dominant polarization of cDC1 to Th1 and CD8^+^ T cell activation rather than IL-23-dependent Th17 induction (30). Two aspects warrant further investigation in future studies. First, our analyses defined Th17 cells solely by IL-17A expression and did not delineate GM-CSF^+^ pathogenic subsets. Second, the cDC1-CD8^+^ T-cell priming assays were conducted with OVA rather than the disease-relevant MOG antigen. Incorporating these elements in subsequent work will refine the mechanistic interpretation.

During recovery, antigen levels decline and TCR signaling weakens (31, 32). However, Regnase-1 reduced cDC1 with maintained a modest yet persistent TCR stimulation and antigen presentation, a signaling pattern that favor memory CD8^+^ T cells differentiation (33). IL-6 accumulation may further influence T cell transcriptional programs by limiting the persistent expression of effector-associated genes while promoting the induction of memory-related transcription factors (34). These mechanistic aspects will need to be further explored in future studies. Additional phenotypic markers beyond CD44^+^CD62L^+^ will be necessary to confirm the Tcm identity, and the precise mechanism by which Regnase-1 loss in cDC1 contributes to Tcm generation remains to be elucidated.

Notably, the poor viability of *Regnase-1^fl/fl^ Xcr1-Cre^+^* mice suggests an essential role of Regnase-1 in maintaining Xcr1^+^ immune populations during development, such as cDC1 and thymic DCs. As *Regnase-1^fl/fl^ CD11c-Cre^+^* mice were viable, this lethality is unlikely due to Cre toxicity (26). Therefore, we used heterozygous mice for our experiments and observed phenotypes may reflect only partial reduction of Regnase-1 and whether complete deficiency leads to more severe immune dysregulation remains to be determined.

This study has several limitations. Our immune profiling was mainly performed at days 16 and 25, and intermediate time points were not analyzed, which may limit a full mechanistic understanding of the biphasic disease pattern. In addition, other myeloid populations such as cDC2, microglia, and macrophages were not systematically examined, and thus indirect effects cannot be excluded. Furthermore, although *Xcr1-Cre* mainly targets cDC1, minor effects on other *Xcr1*-expressing cells cannot be ruled out. Whether the enhanced T-cell activation observed in Regnase-1-reduced cDC1 is directly mediated by increased proinflammatory activity also remains to be clarified. Future studies using mixed bone marrow chimeras will help confirm the cell-autonomous role of Regnase-1 in cDC1.

In conclusion, our study identifies Regnase-1 as a key regulator of cDC1-mediated inflammatory responses. Its reduction accelerates EAE onset, enhancing proinflammatory gene expression and promoting Th1 and CD8^+^ T-cell activation, and later facilitates recovery accompanied by Tcm formation. This dual role highlights the complex function of Regnase-1 in maintaining immune balance. From a translational perspective, targeting Regnase-1 may offer new therapeutic opportunities for autoimmune inflammation, but achieving cell type-specific and phase-dependent modulation without inducing systemic immune activation remains a key challenge.

## Data Availability

The sequencing data generated in this study have been deposited in the DNA Data Bank of Japan (DDBJ) Sequence Read Archive (SRA) under accession number: PRJDB39725.
