# Supplementary material for: Regnase-1 in cDC1 controls T cell priming and shapes the dynamics of experimental autoimmune encephalomyelitis
Source: Front Immunol. 2026 Jan 5;16:1725702. doi: 10.3389/fimmu.2025.1725702 (PMC12812901; doi:10.3389/fimmu.2025.1725702)
Supplement: Supplementary file 1 [file Supplementaryfile1.pdf]

Supplementary Material

1 Supplementary Tables

Table S1. The sequence of the primers for qPCR

Gene	Forward primer	Reverse primer
Actb	GGCTGTATTCCCCTCCATCG	CCAGTTGGTAACAATGCCATGT
Zc3h12a	CGAGAGGCAGGAGTGGAAAC	CTTACGAAGGAAGTTGTCCAGGCTAG
Nfkbiz	GCTCCGACTCCTCCGATTTC	GAGTTCTTCACGCGAACACC
Il1b	GTAGCTATGGTACTCCAGAAGAC	ACGATGATGCACTTGCAGAA
Il6	GCAACTGTTCCTGAACTCAACT	ATCTTTTGGGGTCCGTCAACT
Il12a	CTGTGCCTTGGTAGCATCTATG	GCAGAGTCTCGCCATTATGATTC
Il12b	TGGTTTGCCATCGTTTTGCTG	ACAGGTGAGGTTCACTGTTTCT
Cd86	TGTTTCCGTGGAGACGCAAG	TTGAGCCTTTGTAAATGGGCA
Cd40	TGTCATCTGTGAAAAGGTGGTC	ACTGGAGCAGCGGTGTTATG
Ifit3	GCTCAGGCTTACGTTGACAAGG	CTTTAGGCGTGTCCATCCTTCC
Mx1	GACCATAGGGGTCTTGACCAA	AGACTTGCTCTTTCTGAAAAGCC
Isg15	GGTGTCCGTGACTAACTCCAT	TGGAAAGGGTAAGACCGTCCT

2 Supplementary Figure

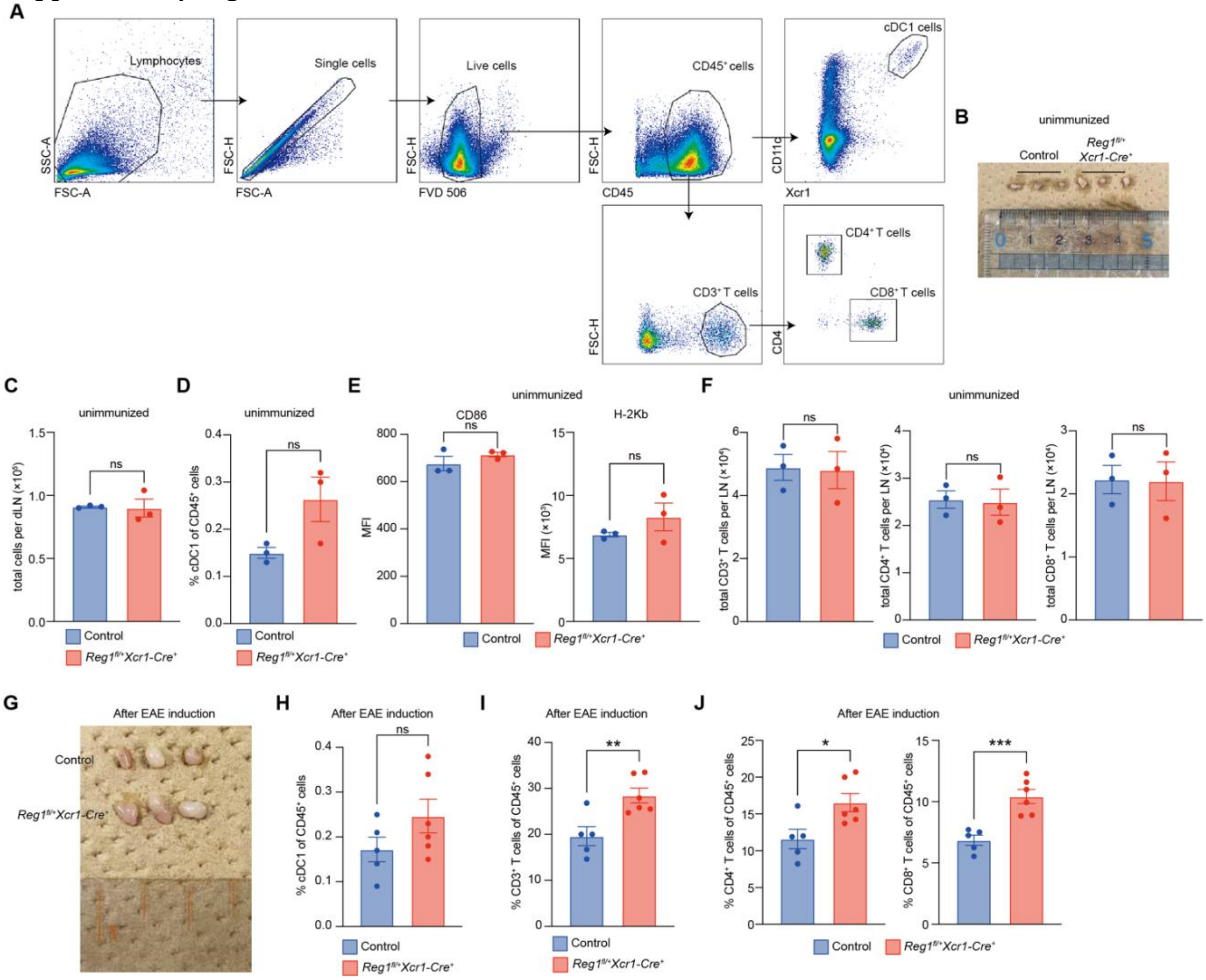

Supplementary Figure 1. related to Figure 4, Reduced expression of Regnase-1 in cDC1 drives peripheral T cell expansion during EAE progression phase

(A) Gating strategy for flow cytometric analysis of LN cells

(B-F) LNs from unimmunized control and *Regnase-1*$^{fl/+}$ *Xcr1-Cre*$^{+}$ mice.

(B) Representative images of LNs.

(C) Total cell numbers per LN.

(D) Frequency of cDC1 among $CD45^{+}$ cells in LN.

(E) Expression of CD86 and H-$2K^{b}$ on cDC1 in LN.

(F) Total numbers of $CD3^{+}$ ($CD45^{+}CD3^{+}$) T cells, $CD4^{+}$ ($CD45^{+}CD3^{+}CD4^{+}$) and $CD8^{+}$ ($CD45^{+}CD3^{+}CD8^{+}$) T cells per LN.

(G-J) DLNs from control and *Regnase-1*$^{fl/+}$ *Xcr1-Cre*$^{+}$ mice at day 16 post-EAE induction.

(G) Representative images of DLNs.

(H) Frequency of cDC1 among $CD45^{+}$ cells in DLN.

(I) Frequency of $CD3^{+}$ T cells among $CD45^{+}$ cells in DLN.

(J) Frequencies of $CD4^{+}$ and $CD8^{+}$ T cells among $CD45^{+}$ cells in DLN.

Data are presented as mean ±s.e.m. Statistical analyses were performed using unpaired t test. DLN, draining lymph node; Reg1, Regnase-1. *, $P < 0.05$; ***, $P < 0.001$, ns, not significant.

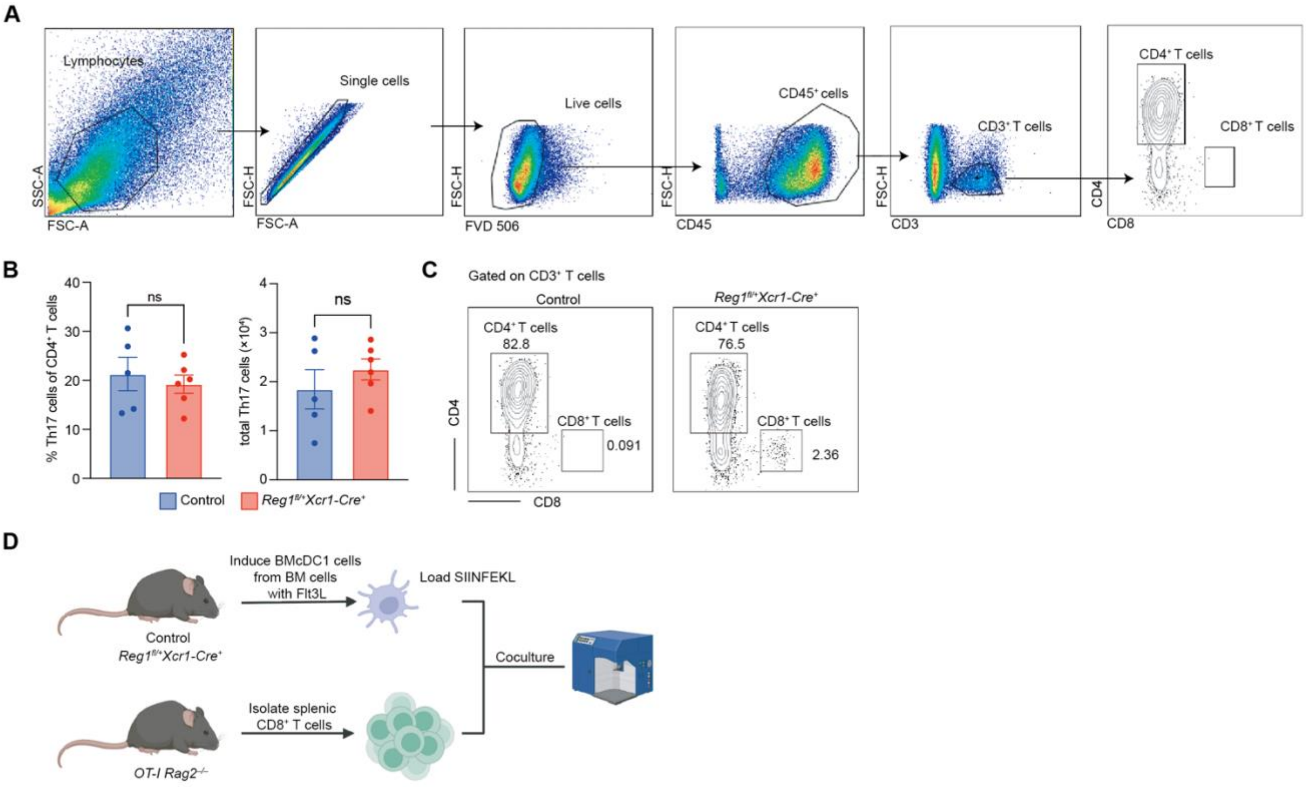

Supplementary Figure 2. related to Figure 5, Reduced Regnase-1 expression in cDC1 promotes CD8$^+$ T cell infiltration and effector function in the CNS

(A) Gating strategy for identifying infiltrating immune cells from the spinal cord of EAE mice.

(B) Frequency and total number of Th17 cells (CD45$^+$CD3$^+$CD4$^+$ IL-17A$^+$ IFN-γ^-) among spinal cord-infiltrating CD4$^+$ T cells.

(C) Representative plots showing spinal cord-infiltrating CD4$^+$ and CD8$^+$ T cells.

(D) Schematic of the in vitro co-culture assay. BM-cDC1s from *Regnase-1*$^{fl/+}$ *Xcr1-Cre*$^+$ or control mice were loaded with SIINFEKL peptide, and co-cultured with splenic CD8$^+$ T cells isolated from OT-I Rag2$^{-/-}$ mice for 48 hours (for CellTracer proliferation test) and 24 hours (for cytokine detection) before flow cytometry analysis.

Data are presented as mean ±s.e.m. Statistical analyses were performed using unpaired t test. DLN, draining lymph node; Reg1, Regnase-1; BM-cDC1, bone marrow-derived conventional DC1; ns, not significant.
